# Supplementary material for: Adipocyte metabolic state regulates glial phagocytic function
Source: bioRxiv. 2024 Sep 24:2024.09.24.614765. Preprint. [Version 1] doi: 10.1101/2024.09.24.614765 (PMC11463506; doi:10.1101/2024.09.24.614765)
Supplement: Supplement 1 [file media-1.pdf]

Figure S1. Adipocyte glycolytic breakdown occurs before it does in the brain.

A) Confocal images of Ldh-GFP in the adipose tissue of flies fed either an ND or an HSD for 2 weeks.

B) Mean Ldh-GFP fluorescent intensity, obtained from Z-stack summation projections of adipose tissue in ND and HSD-fed flies. Student's t-test with Welch's correction. N = each circle represents an individual fly.

C) Timeline of Ldh reduction in adipose tissue versus the brain on HSD. Ldh is downregulated in adipose tissue prior to its downregulation in the brain.

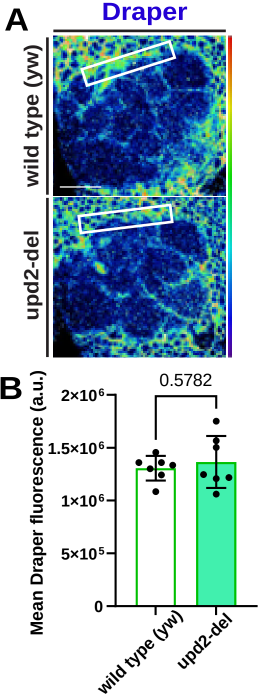
A
Draper
wild type (yw)
upd2-del
B
Mean Draper fluorescence (a.u.)
2×10⁶
1.5×10⁶
1×10⁶
5×10⁵
0
0.5782
wild type (yw)
upd2-del

Figure S2. Adipocyte glycolytic breakdown occurs before it does in the brain.

Mean beta-hydroxybutyrate in the hemolymph flies fed either an ND or an HSD for 1 week. Student's t-test with Welch's correction. N = 3 technical replicates of 30 flies/replicate.

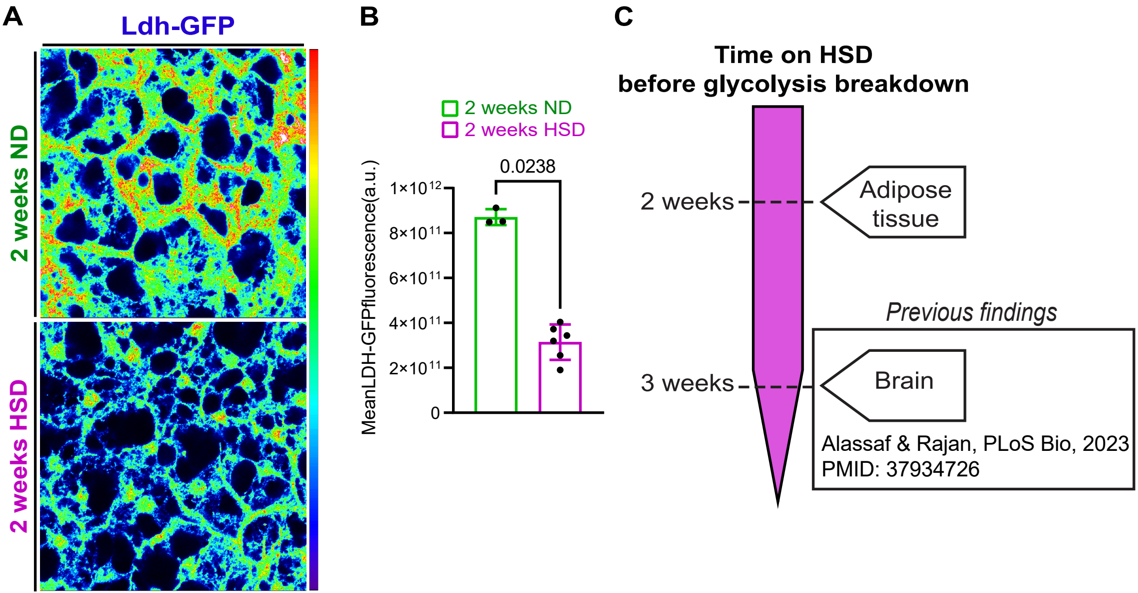

A
Ldh-GFP
2 weeks ND
2 weeks HSD
B
2 weeks ND
2 weeks HSD
0.0238
MeanLDH-GFPfluorescence(a.u.)
1×10^12
8×10^11
6×10^11
4×10^11
2×10^11
0
C
Time on HSD
before glycolysis breakdown
2 weeks
Adipose tissue
Previous findings
3 weeks
Brain
Alassaf & Rajan, PLoS Bio, 2023
PMID: 37934726

Figure S3. Upd2 does not affect Draper levels in the ensheathing glia.

A) Confocal images of the antennal lobe region of wild type and upd2-deletion flies immunostained with anti-Draper.

B) The mean fluorescent intensity of Draper was measured within a region of interest (white box) that coincided with the location of the ensheathing glia. These measurements were derived from a Z-stack summation projection covering the entire depth of the antennal lobe. Student t-test with Welch's correction. N = each circle represents an individual fly.

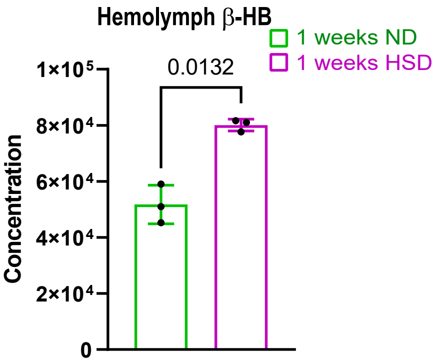

Hemolymph β-HB
1 weeks ND
1 weeks HSD
0.0132
1×10^5
8×10^4
6×10^4
4×10^4
2×10^4
0
Concentration

Figure S4. HSD treatment reduces hemolymph PE levels.

A) The mean concentration of phosphatidylethanolamine (PE) in the hemolymph of flies fed either an ND or a HSD for 2 weeks. N = 3 technical replicates of 30 flies/replicate.

B) The mean composition of phosphatidylethanolamine (PE) in the hemolymph of flies fed either an ND or a HSD for 2 weeks. N = 3 technical replicates of 30 flies/replicate.

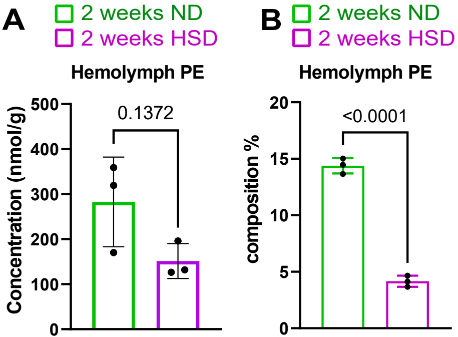
A
2 weeks ND
2 weeks HSD
Hemolymph PE
Concentration (nmol/g)
0.1372
0
100
200
300
400
500
B
2 weeks ND
2 weeks HSD
Hemolymph PE
composition %
<0.0001
0
5
10
15
20

Figure S5. Knocking down ApoB in adipose tissue does not impact insulin levels in the brain.

A) Z-stack summation projections of the insulin-producing cells (IPC) immunostained with anti-Dilp5 of flies expressing either Luciferase-RNAi or ApoB-RNAi specifically in their adipose tissue.

B) Mean fluorescent intensity of Dilp5 derived from a Z-stack summation projection covering the entire depth of the IPCs. Student t-test with Welch's correction. N = each circle represents an individual fly.

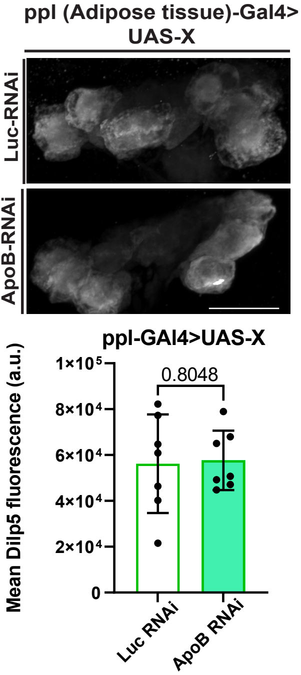
ppl (Adipose tissue)-Gal4>
UAS-X
Luc-RNAi
ApoB-RNAi
ppl-GAl4>UAS-X
Mean Dilp5 fluorescence (a.u.)
1×10^5
8×10^4
6×10^4
4×10^4
2×10^4
0
0.8048
Luc RNAi
ApoB RNAi
